# A meta‐analysis on allergen‐specific immunotherapy using MCT^®^ (MicroCrystalline Tyrosine)‐adsorbed allergoids in pollen allergic patients suffering from allergic rhinoconjunctivitis

**DOI:** 10.1002/clt2.12037

**Published:** 2021-06-03

**Authors:** Sven Becker, Petra Zieglmayer, Gabriela Canto, Filippo Fassio, Patrick Yong, Cengizhan Acikel, Esther Raskopf, Esther Helen Steveling‐Klein, Silke Allekotte, Ralph Mösges

**Affiliations:** ^1^ Department of Otorhinolaryngology, Head and Neck Surgery University of Tübingen Tübingen Germany; ^2^ Karl Landsteiner University Krems Austria; ^3^ Vienna Challenge Chamber Power Project GmbH Vienna Austria; ^4^ Hospital Infanta Leonor Madrid Spain; ^5^ Allergy and Clinical Immunology Unit Azienda Usl Toscana Centro, Ospedale San Giovanni di Dio Florence Italy; ^6^ Department of Clinical Immunology and Allergy Frimley Park Hospital Frimley UK; ^7^ Royal Surrey County Hospital Guildford UK; ^8^ ClinCompetence Cologne GmbH Cologne Germany; ^9^ Dermatology Department, Division of Allergy University Hospital Basel Basel Switzerland; ^10^ Institute of Medical Statistics and Computational Biology, Faculty of Medicine University of Cologne Cologne Germany

**Keywords:** adjuvanz, allergenimmuntherapie, allergische rhinokonjunktivitis, allergoid, Mikrokristallines Tyroson (MCT^®^)

## Abstract

**Background:**

The World Allergy Organization and the European Academy of Allergy and Clinical Immunology recommend to perform product‐specific meta‐analyses for allergen‐specific immunotherapies because of the high degree of heterogeneity between individual products. This meta‐analysis evaluates the efficacy and safety of Glutaraldehyde‐modified and MCT^®^ (MicroCrystalline Tyrosine)‐adsorbed allergoids (MATA).

**Methods:**

The databases MEDLINE, LILACS, embase, LIVIVO, Web of Science and Google (Scholar) were searched for publications on MATA up to June 2019. Primary endpoint was the combined symptom and medication score (CSMS). Secondary endpoints were single scores, immunogenicity and improvement of allergic condition. Secondary safety endpoints were the occurrence of side effects. A random effects model was applied with (standardized) mean differences ([S]MDs) including confidence intervals (CI). Heterogeneity was analyzed using the I^2^ index and publication bias using Egger's test and Funnel plots. Subgroups were analyzed regarding age and asthma status.

**Results:**

Eight randomized double‐blind placebo‐controlled trials were selected for efficacy and 43 publications for safety analysis. In total, 4531 patients were included in this analysis including eight studies containing data on children and adolescents. AIT with MATA significantly reduced allergic symptoms and medication use with a SMD for CSMS of −0.8 (CI: −1.24, −0.36) in comparison to placebo. Heterogeneity was moderate between the studies. The total symptom score (−1.2 [CI: −2.11, −0.29]) and the total medication score (−2.2 [CI: −3.65, −0.74]) were also significantly reduced after MATA treatment. Patient's condition improved significantly after treatment with MATA, with an odds ratio of 3.05 (CI: 1.90, 4.90) when compared to placebo. The proportion of patients, who developed side effects was 38% (CI: 19%, 57%). No serious side effects occurred. Safety in the subgroups of asthmatic patients, children and adolescents did not differ from the overall patient population.

**Conclusions:**

This meta‐analysis reveals a large body of evidence from publications investigating MATA. MATA significantly improved allergic symptoms and reduced the use of anti‐allergic medication in comparison to placebo, with an excellent safety profile. Especially for children and asthmatic patients, the use of MATAs can be considered as safe, because the safety profiles in these groups did not differ from the total patient population.

## 1 BACKGROUND

Meta‐analyses provide a systematic and statistically validated overview and combine the results of earlier studies; therefore, broadening and rating the evidence for the investigated body of research.^1^ For example, a meta‐analysis on allergen immunotherapy for allergic rhinoconjunctivitis by Dhami and colleagues gives such an overview but lacks product‐specific details.^2^ This may be sufficient in many areas of research, but in some cases, like treating patients with allergic rhinoconjunctivitis, the published meta‐analysis lacks specificity because of the heterogeneity of the analyzed studies and diversity of included products. To overcome this, the World Allergy Organization (WAO) published a statement^3^ in which they propose to conduct product‐specific meta‐analyses to establish clinical efficacy for individual products. Also, EAACI (European Academy of Allergy and Clinical Immunology) recommended in their allergen immunotherapy (AIT) guideline^4^ a product‐specific evaluation as a reasonable approach. Since the products differ considerably with regard to allergen‐concentrations, modifications, adjuvants, and dosing schemes, the results obtained from one product cannot be extrapolated to other products or be generalized. Because of their different structure, adjuvants, and adsorbents, modified allergen extracts like MicroCrystalline Tyrosine (MCT^®^)‐adsorbed allergoids cannot be compared with, for example, aqueous native allergen extracts.

The allergen extracts analyzed here are glutaraldehyde‐modified (so called allergoids), as well as MCT^®^‐adsorbed. These MATAs (glutaraldehyde‐modified and MCT^®^‐adsorbed allergoids), have been marketed already since the early 1970s. The modification of allergen extracts with glutaraldehyde was first described by Patterson and co‐workers.^5^ The modification with glutaraldehyde cross‐links the proteins, resulting in high molecular weight allergen polymers. These inhibit IgE binding, leading to a reduced allergenicity, but do not decrease immunogenicity.^6^ Because of the reduced allergenicity, the updosing can be done much faster without compromising safety.^7^, ^8^ However, allergoids are rarely applied without adjuvants like aluminium or MCT^®^. MCT^®^ acts as a depot‐adjuvant facilitating a slow release of the allergen extract,^9^ it is biodegradable with a half‐life of 48 h at the injection‐site and a strong Th1 polarizing adjuvant without known safety concerns.^9^ This short half‐life in the tissue (biodegradability) and its biocompatibility indicates a favorable role as adjuvant compared to aluminium.^10^ When looking especially at clinical trials, 1575 patients in placebo arms received MCT^®^ alone, without reporting any serious adverse events with regards to the treatment.^11^ Also, a recent head‐to‐head comparison confirmed the superior safety profile of MCT^®^ when compared with aluminium.^12^

The various MATA preparations include, for example, either a grass mix, a tree mix, ragweed or other weeds like plantain and/or mugwort. Many clinical trials, including randomized controlled trials (RCT) and double‐blind placebo‐controlled trials (DBPCT) demonstrated that MATAs are effective, safe, and well tolerated.^11^, ^13^, ^14^ MATAs are licensed as a pre‐seasonal and perennial treatment.

Here, we performed a systematic review and meta‐analysis of studies conducted with the product‐line of MATAs to compare the efficacy and safety with the current generalized meta‐analysis by Dhami et al. and other product‐specific analyses.

## 2 METHODS

### 2.1 Search strategy

An extensive search was conducted in the following databases: MEDLINE, LILACS, LIVIVO, and Web of Science. In addition, literature was searched using Google and Google Scholar. Search terms were “desensitization,” “MATA,” “tyrosine allergoid,” and “glutaraldehyde‐modified tyrosine adsorbate,” including various combinations of these search terms (Additional file 1). Bencard Allergie GmbH has also provided the authors with a list of publications referring to studies utilizing MATAs.

Literature was included in this systematic review up to June 12, 2019. There were no language restrictions. Where applicable, relevant literature was translated into English. If full publications were not available online, they were requested (if possible) from the first author.

### 2.2 Inclusion and exclusion criteria

For this meta‐analysis, all publications were considered in which patients with allergic rhinoconjunctivitis (with or without asthma) were treated with MATA, independent of applied dose and treatment/study duration. Age was not restricted and studies with children and adolescents were also included. Studies using the following MATA allergens were included: tree pollen with or without other allergens, grass pollen mix with or without other allergens as well as ragweed with or without other allergens (Table 1). Regarding study design, DBPCTs, randomized controlled trials, controlled trials and uncontrolled studies that were either conducted prospectively or retrospectively, were included in the analysis. For the analysis of the efficacy endpoints (primary and secondary), only DBPCTs were included, because the blinding ensures an unbiased conduct of the studies.

**TABLE 1:** Overview of the MATA allergens included in this meta‐analysis with current tradenames of the respective market areas

Allergen(s)	Tradename	Market area
Birch/Alder/hazel (1:1:1)	POLVAC™ Bäume	Switzerland
	Pollinex Boom	Netherlands
	Pollinex trees	United Kingdom, Belarus, Macedonia, Serbia, Croatia
	TA Bäume top	Germany
	POLLINEX tree	Czech Republic, Poland, Slovakia, Estonia, Latvia, Lithuania
	Pollinex tree combi	Albania
Tree mixes	POLLIGOID^®^	Spain
	M.A.T.A. PFS tree pollen mix	Italy
12‐Grass mix	Pollinex Graspollen	Netherlands
	M.A.T.A Graminacee	Italy
12‐Grass mix + rye	Pollinex grasses + Rye	United Kingdom, Belarus, Macedonia, Serbia, Croatia
	POLLIGOID^®^	Spain
	POLLINEX Rye	Czech Republic, Slovakia, Estonia, Latvia, Lithuania
	Pollinex + Rye	Poland
	TA Gräser top	Germany
	POLVAC™ Gräser + Roggen	Switzerland
	Pollinex grass combi	Albania
Ragweed	Pollinex‐R	Canada, Slovakia
Weed mixes	POLLIGOID^®^	Spain
	TA Kräuter top	Germany, Croatia

Reviews, case reports, animal studies and studies containing data on aluminium absorbed allergen extracts were excluded.

### 2.3 Endpoints

The primary endpoint was the combined symptom and medication score (CSMS) in randomized DBPCTs using MATA. The analysis was conducted similar to the primary analysis in the original studies.^14^, ^15^, ^16^ In the two‐year studies, only the first year of treatment was considered. Covariates with regard to the primary endpoint were responders and non‐responders as defined in the respective studies.^14^, ^15^, ^16^, ^17^, ^18^

Secondary endpoints (also analyzed in the DBPCTs) were the total symptom score (TSS) including the individual symptoms sneezing, nasal obstruction, eye symptoms and cough,^14^, ^15^, ^16^, ^19^ the total medication score (TMS)^14^, ^15^, ^16^, ^20^ as well as immunologic parameters (allergen‐specific Immunoglobulin G, sIgG).^13^, ^14^, ^16^

Safety was assessed by analyzing the occurrence of solicited local and systemic adverse events as well as other adverse reactions with regard to the number of patients and the number of injections in the included DBPCTs, randomized controlled trials, controlled trials and uncontrolled studies.

### 2.4 Data extraction and analysis

The data analyzed here were extracted from the publications by two different reviewers in Excel files. In case of discrepancies, this issue was discussed with the co‐authors until a consensus was reached. The means and standard deviations (SD) of the CSMS, the TMS, the TSS, as well as the single symptom scores were extracted from the DBPCTs.

For the analysis of the total symptom score (TSS) all studies were used that provided results on nasal symptoms (sneezing, rhinorrhoea/congestion/nasal obstruction), eye symptoms, and coughing.^14^, ^15^, ^16^, ^19^ For the total medication score, all studies with data on symptomatic medication were included (use of antihistamines, beclomethasone, chlorpheniramine, etc.).^14^, ^15^, ^16^, ^20^ The single scores were then added up to the TSS or TMS, respectively. If the data was not expressed in a daily score but for the whole pollen season/observation period, the scores were converted by dividing the TSS or TMS by the days of the pollen season/observation period defined in the publications. The combined symptom and medication score (CSMS) was then generated by adding the TSS and TMS of the respective study.^14^, ^15^, ^16^

To evaluate the treatment effect (improvement of allergic condition), a subgroup analysis was performed, where the number of responders in the treatment and placebo groups was analyzed. For this, the definition of responders was taken from the respective studies:^14^, ^15^, ^16^, ^17^, ^18^ responders were defined as patients with improved symptoms after treatment or who had at least a good response to the treatment. Non‐responders were defined as patients who had no change or even a deterioration, or who had a poor response to the treatment. The evaluation was either done by the patients themselves, by the parents of the patients or by the physician (for details on the categorization, please refer to Additional file 2).

For the DBPCTs, data about safety were extracted for the treatment and the placebo groups as the number of immediate and late local reactions per injection, as well as systemic reactions per patient and per injection. Data on safety was not only extracted from the DBPCTs, but also from other (uncontrolled) studies which collected data on local and systemic reactions. For these studies, data were extracted as the number of local reactions per patient or per injection and as the number of systemic reactions per patient or per injection. Furthermore, data on patients with adverse drug reactions in relation to the overall number of patients in the respective study were extracted. The methodological quality of the DBPCTs was evaluated by two independent reviewers using the Jadad scoring system.^21^

The analyses were performed in RevMan 5 (Version 5.3, The Cochrane Collaboration, London, UK, 2020) and presented as Forest plots and Funnel plots (for publication bias). Reporting of the meta‐analysis was done according to the PRISMA statement^22^ and the checklist was completed (Additional file 3).

### 2.5 Statistical analysis

Random effects modeling was applied for meta‐analysis if it was clinically or statistically suitable. The inverse variance (IV) method was applied for study weights. As summary statistics, the standardized mean difference (SMD) was used for results of a continuous outcome or odds/risk ratio (OR/RR), for results of a binary outcome. Where applicable, a confidence interval (CI) of 95% was included.

#### 2.5.1 Analysis of heterogeneity and publication bias

Heterogeneity was identified with the I^2^ test. With regard to the I^2^ test, an I^2^ value <25% was considered as homogenous between the analyzed studies. A I^2^ value of 26%–50% would identify a low heterogeneity and 51%–75% a moderate heterogeneity between studies. An I^2^ value >75% was defined as high heterogeneity.^23^ Comparison of means between studies was done with the Z‐test.

To test for publication bias, funnel plots (with 95% CI, where applicable) were created for the primary and secondary efficacy outcomes^24^ and tested with the Egger’s regression test^24^ and the Begg’s rank correlation test (Additional [Link], [Link], [Link]).

#### 2.5.2 Subgroup analysis

In addition, subgroup analyses were performed with regard to the safety of MATAs in vulnerable groups like children and adolescents, as well as asthmatics.

The publications were checked for inclusion of data on patients <18 years. Studies with a mixed patient collective (children/adolescents and adults), or only adults were also analyzed for comparison. Studies, where the age‐range of the patients was not given, were included in the NK (not known) group. Similarly, the publications were checked for patients with asthma. Studies including both asthmatic and non‐asthmatic patients, as well as studies only including non‐asthmatics were evaluated for comparison. Studies, where this was not documented were included in the NK (not known) group.

## 3 RESULTS

### 3.1 Systematic review

Two hundred sixty‐nine publications were found after database and Google search. After removing duplicates (*N* = 117), 152 publications remained. Of those, 25 were excluded, because the full text was not available, the publications were case reports, reviews, surveys, or publications presenting additional data of studies that were already included. This resulted in 127 publications.

With regard to the safety aspect, 52 out of the 127 were excluded because data on safety was documented, but MATA was not used in these studies. Another 32 publications were excluded because no safety data was documented. This resulted in 43 studies^13^, ^14^, ^15^, ^16^, ^17^, ^20^, ^25^, ^26^, ^27^, ^28^, ^29^, ^30^, ^31^, ^32^, ^33^, ^34^, ^35^, ^36^, ^37^, ^38^, ^39^, ^40^, ^41^, ^42^, ^43^, ^44^, ^45^, ^46^, ^47^, ^48^, ^49^, ^50^, ^51^, ^52^, ^53^, ^54^, ^55^, ^56^, ^57^, ^58^, ^59^, ^60^, ^61^ which were included in the safety analysis, with 6 DBPCTs, 6 randomized controlled trials (RCTs), 6 controlled trials (CTs), and 25 non‐controlled studies. For the subgroup analysis of children/adolescents^15^, ^17^, ^25^, ^26^, ^28^, ^29^, ^30^, ^31^, ^33^, ^35^, ^36^, ^37^, ^38^, ^40^, ^42^, ^44^, ^45^, ^46^, ^49^, ^51^, ^54^, ^55^, ^56^, ^57^, ^58^, ^59^, ^60^ and asthmatic patients,^13^, ^14^, ^16^, ^27^, ^29^, ^30^, ^32^, ^34^, ^35^, ^37^, ^38^, ^40^, ^41^, ^42^, ^43^, ^44^, ^47^, ^49^, ^50^, ^51^, ^52^, ^53^, ^54^, ^55^, ^56^, ^57^, ^61^ 27 publications were identified and included, respectively.

**FIGURE 1:**
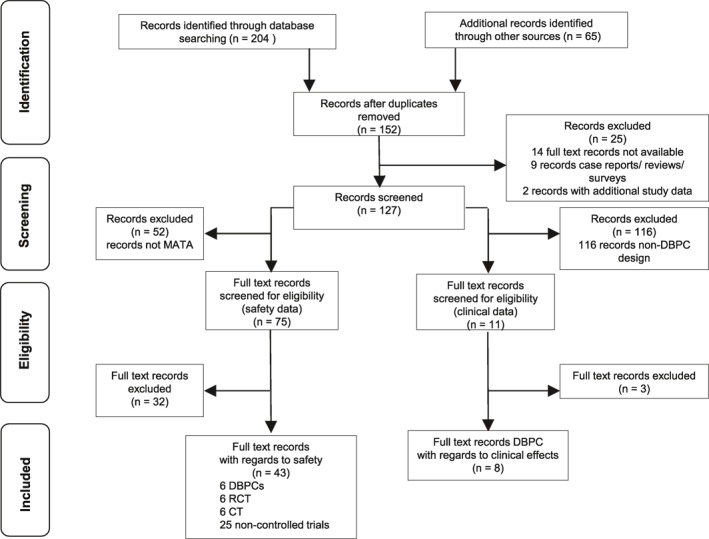
PRISMA flow chart depicting the selection process for studies investigating the treatment with MATA

For the analysis of the clinical endpoints, 116 of the 127 studies were not DBPC. Another 3 of the remaining 11 studies were excluded because data on symptom or medication intake during the season were not documented. Finally, eight publications^13^, ^14^, ^15^, ^16^, ^17^, ^18^, ^19^, ^20^ fulfilled the study selection criteria (DBPC) with regard to clinical effects (Figure 1).

The DBPCTs were also analyzed regarding methodological quality: six of the eight studies gained three score points or more,^13^, ^14^, ^15^, ^18^, ^19^, ^20^ of which three studies scored five points.^13^, ^14^, ^18^ Two studies (both from 1979) gained two points.^16^, ^17^ Details on the distribution of the score points are displayed in Table 2.

**TABLE 2:** Study characteristics and Jadad score of the double‐blind placebo‐controlled trials

Publication reference year	Allergen	Patient group	Indication	Treatment schedule	Cumulative dose	*N* patients total	*N* patients MATA	*N* patients placebo	Jadad‐score			
									Randomization	Blinding	Drop‐outs	Total
Adamek‐Guzik^17^ 1979	12 grass mix	Children + adults	Hayfever ± AA	3 injections 300/800/2000 NU	3100 NU	223	170	53	0	2	0	2
Cockroft^14^ 1977	Ragweed	Unknown	AR	4 injections 300/700/2000/4000 NU	7000 NU	43	21	22	2	2	1	5
Kuna^19^ 1989	12 grass mix	Unknown	AA	3 injections 300/800/2000 NU	3100 NU	24	12	12	1	2	NA^a^	3^a^
Metzger^13^ 1981	Ragweed	Unknown	Hayfever	5 injections 350/1000/3000/6000/6000 NU	16 350 NU	100	52	48	0	2	1	3
Miller^18^ 1974	12 grass mix	Unknown	Hayfever	9 injections 25/50/100/250/500/1000/2500/5000/10 000 NU	19 425 NU	67	35	22	2	2	1	5
Mischler^15^ 1981	Ragweed	Children + adults	AR	4 injections 300/700/2000/6000 NU	9000 NU	366	177	189	2	2	1	5
Roznieka^20^ 1995	Grass	Unknown	Pollinosis	3 injections 300/800/2000 NU	3100 NU	27	14	13	2	2	0	4
Weisnagel^16^ 1979	Ragweed	Children	ARC +/− AA	4 injections 300/700/2000/6000 NU	9000 NU	34	17	17	0	2	0	2

Abbreviations: AA, allergic asthma; AR, allergic rhinitis; ARC, allergic rhinoconjunctivitis; NU, Noon Units.

^a^ None of the patients dropped out from this study.

### 3.2 Study characteristics and patient population

All DBPCTs which were considered for analysis were published between 1974 and 1995. The studies included 884 patients with hay fever or seasonal allergic rhinoconjunctivitis with or without allergic asthma. Of those, 498 patients were treated with allergen extracts containing either a grass mix,^17^, ^18^, ^19^, ^20^ or ragweed.^13^, ^14^, ^15^, ^16^ Treatment schedules ranged from three to nine injections with cumulative doses of up to 19 425 Noon Units (Table 2).

The studies which included safety data were published from 1974 to 2003. In total, 43 publications with 4531 patients were analyzed. Some of the analyzed studies also comprised data on children and adolescents: eight studies clearly documented the number of included children and adolescents (258 in total). Three studies did not include children and adolescents. Thirty studies did not state the age‐group or the number of included children and adolescents.

### 3.3 Primary efficacy analysis: combined symptom and medication score CSMS

**FIGURE 2:**
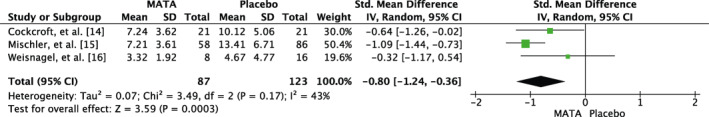
Meta‐analysis of the DBPCTs comparing the combined symptom and medication score (CSMS) of patients treated with MATA and placebo treated patients at the time of the primary analysis in the respective studies. The random effects model was applied with inverse variance (IV) for study weight. Results are displayed as standardized mean difference with 95% CI (confidence interval) as well as analysis of heterogeneity. The studies are presented with N patients, mean and SD (standard deviation)

Of the eight randomized DBPCTs included in this meta‐analysis, three studies^14^, ^15^, ^16^ were evaluable with regard to the CSMS (*N* = 210 patients): with application of the random effects model, the SMD was −0.80 (CI: −1.24; −0.36) for patients treated with MATA in comparison to the placebo group. The test for heterogeneity revealed an I^2^ of 43%, showing low heterogeneity between the studies. The test for overall effects revealed a *Z* of 3.59 (*p* = 0.0003, Figure 2).

### 3.4 Secondary efficacy analysis: total (TSS, TMS) and single scores

**FIGURE 3:**
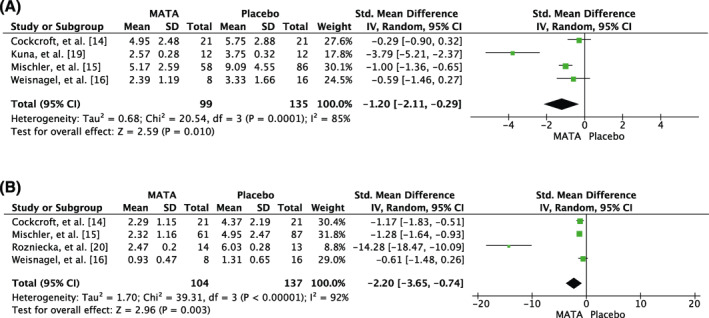
Meta‐analysis of the DBPCTs comparing the (A) total symptom score (TSS) and (B) total medication score (TMS) of patients treated with MATA and placebo treated patients at the time of the primary analysis in the respective studies. The random effects model was applied with inverse variance (IV) for study weight. Results are displayed as standardized mean difference with 95% CI (confidence interval) as well as analysis of heterogeneity. The studies are presented with N of patients, mean and SD (standard deviation)

The TSS was evaluable in 4 DBPCTs with 234 patients:^14^, ^15^, ^16^, ^19^ using the random effects model, the SMD was −1.20 (CI: −2.11; −0.29), when comparing MATA treatment with placebo. However, the heterogeneity was high between the studies with an I^2^ of 85%. The test for overall effects (*Z*) was 2.59 when comparing the means of the studies (*p* = 0.01, Figure 3A).

Four studies^14^, ^15^, ^16^, ^20^ were analyzed with regard to the TMS (*N* = 241 patients): with application of the random effects model, the SMD was −2.20 (CI: −3.65; −0.74) for patients treated with MATA in comparison to placebo treated patients. The test for heterogeneity showed a very high heterogeneity between the studies with an I^2^ of 92%. Testing for the overall effect resulted in *Z* = 2.96 (*p* = 0.003), Figure 3B).

Analysis of the single symptom scores (Additional file 4) showed for the symptom sneezing (evaluated in four studies^14^, ^15^, ^16^, ^20^) (*N* = 241 patients) a SMD of −0.51 (CI: −1.01; 0.0), and an I^2^ of 62% (*p* = 0.05). The *Z* test resulted in 1.96 (*p* = 0.05). The SMD for nasal obstruction (analyzed in four studies^14^, ^15^, ^16^, ^20^) (*N* = 236 patients), was −0.52 (CI: −0.79; 0.26), with I^2^ = 0% (*p* = 0.73) and an overall effect *Z* of 3.87 (*p* = 0.0001). The effect of MATA on eye symptoms was analyzed in three studies^14^, ^15^, ^16^ (*N* = 214 patients): the SMD was −0.82 (CI: −1.34; 0.29), with I^2^ = 58% (*p* = 0.09) and a *Z* of 3.05 (*p* = 0.002). The symptom coughing (analyzed in three studies,^14^, ^15^, ^16^ *N* = 214 patients) had a SMD of −0.30 (CI: −1.25; 0.66), with I^2^ = 88% (*p* = 0.0003). The overall effect *Z* was 0.60 (*p* = 0.55).

### 3.5 Secondary efficacy analysis: immunological parameter (sIgG)

**FIGURE 4:**
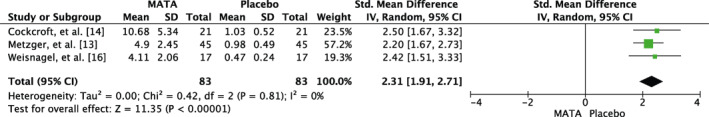
Meta‐analysis of the DBPCTs comparing the results of allergen specific IgG of patients treated with MATA and placebo treated patients at the time of the primary analysis in the respective Studies. The random effects model was applied with inverse variance (IV) for study weight. Results are displayed as standardized mean difference with 95% CI (confidence interval) as well as analysis of heterogeneity. The studies are presented with N of patients, mean and SD (standard deviation)

In three DBPCTs with 166 patients,^14^, ^15^, ^16^ immune responses were also analyzed by determining sIgG. In these studies, the SMD was 2.31 (CI: 1.91; 2.71), showing a strong increase in sIgG following MATA treatment when compared to placebo. Here, I^2^ was 0% revealing a very homogeneous distribution between the studies. The test for the overall effect was *Z* = 11.35 (*p* < 0.00,001, Figure 4).

### 3.6 Secondary efficacy analysis: improvement of allergic condition

**FIGURE 5:**
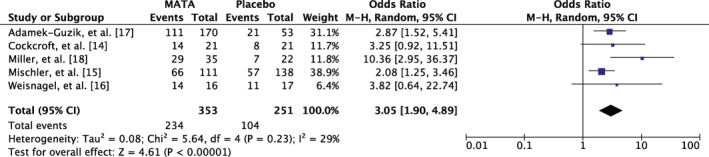
Meta‐analysis of the DBPCTs with regard to the improvement of the allergic condition of patients treated with MATA and placebo treated patients at the time of the primary analysis in the respective studies. The random effects model was applied using the Matel‐Haenszel method (M‐H). Results are displayed as odds ratio (OR) with 95% CI (confidence interval) as well as analysis of heterogeneity. The studies are presented with N of patients, and N of events (= patients who improved after treatment with MATA)

The treatment effect (improvement of allergic condition) was analyzed in five DBPC studies^14^, ^15^, ^16^, ^17^, ^18^ and evaluated by either the patients, parents of the patients or investigators in the respective studies (*N* = 604 patients). The OR was 3.05 (CI: 1.90; 4.89) with I^2^ of 29% displaying a low heterogeneity between the studies. The test for overall effects revealed a *Z* of 4.61 (*p* < 0.00,001), showing that treatment with MATA significantly improved the allergic condition of the patients, while this was not the case for placebo‐treated patients (Figure 5).

### 3.7 Safety analysis

No serious adverse event and no fatalities were documented in the studies identified for the safety analysis. Side effects were documented in 25 studies^15^, ^17^, ^26^, ^28^, ^29^, ^30^, ^31^, ^33^, ^35^, ^36^, ^37^, ^38^, ^40^, ^42^, ^45^, ^46^, ^49^, ^51^, ^54^, ^55^, ^56^, ^57^, ^58^, ^59^, ^60^ and revealed a proportion of 38% (CI: 19%, 57%) of the patients under MATA treatment, and an I^2^ of 100%. The analysis of local reactions per patient was done in 27 studies^15^, ^17^, ^25^, ^26^, ^28^, ^29^, ^30^, ^31^, ^33^, ^35^, ^36^, ^37^, ^38^, ^40^, ^42^, ^44^, ^45^, ^46^, ^49^, ^51^, ^54^, ^55^, ^56^, ^57^, ^58^, ^59^, ^60^ and revealed a proportion of 34% (CI: 23%, 45%) with a heterogeneity I^2^ of 99%. Systemic reactions in the 20 analyzable studies^15^, ^17^, ^25^, ^26^, ^28^, ^29^, ^31^, ^35^, ^37^, ^38^, ^40^, ^42^, ^45^, ^46^, ^49^, ^55^, ^56^, ^58^, ^59^, ^60^ revealed a proportion of 10% (CI: 7%, 13%) but with a high heterogeneity I^2^ of 89%.

Focusing on the DBPCTs (Additional file 5), the RR for the occurrence of immediate local reactions after an injection was 11.61 (CI: 2.09; 64.59), showing an 11‐times higher risk of developing immediate local reactions after treatment with MATA in comparison to placebo: 70.5% of the MATA injections resulted in immediate local reactions and 9.9% of the placebo injections. Here I^2^ was 69%, showing a moderate heterogeneity between the two studies. Late local reactions occurred after 65.1% of MATA injections and after 16.6% of the placebo injections with a RR of 3.51 (CI: 1.68; 7.34). Heterogeneity I^2^ was 88%. Of note, only two studies (*N* = 134 patients and *N* = 606 injections) were included in the analysis of immediate and late local reactions.^13^, ^16^ Systemic reactions (analyzed in three publications with 177 patients receiving 768 injections) occurred after 1.9% of MATA injections, whereas in the placebo group, no systemic reaction occurred after the injections. The RR was 6.43 (CI: 1.14; 36.30) with an I^2^ of 0%.^13^, ^14^, ^16^

### 3.8 Subgroup analysis

#### 3.8.1 Children and adolescents

**FIGURE 6:**
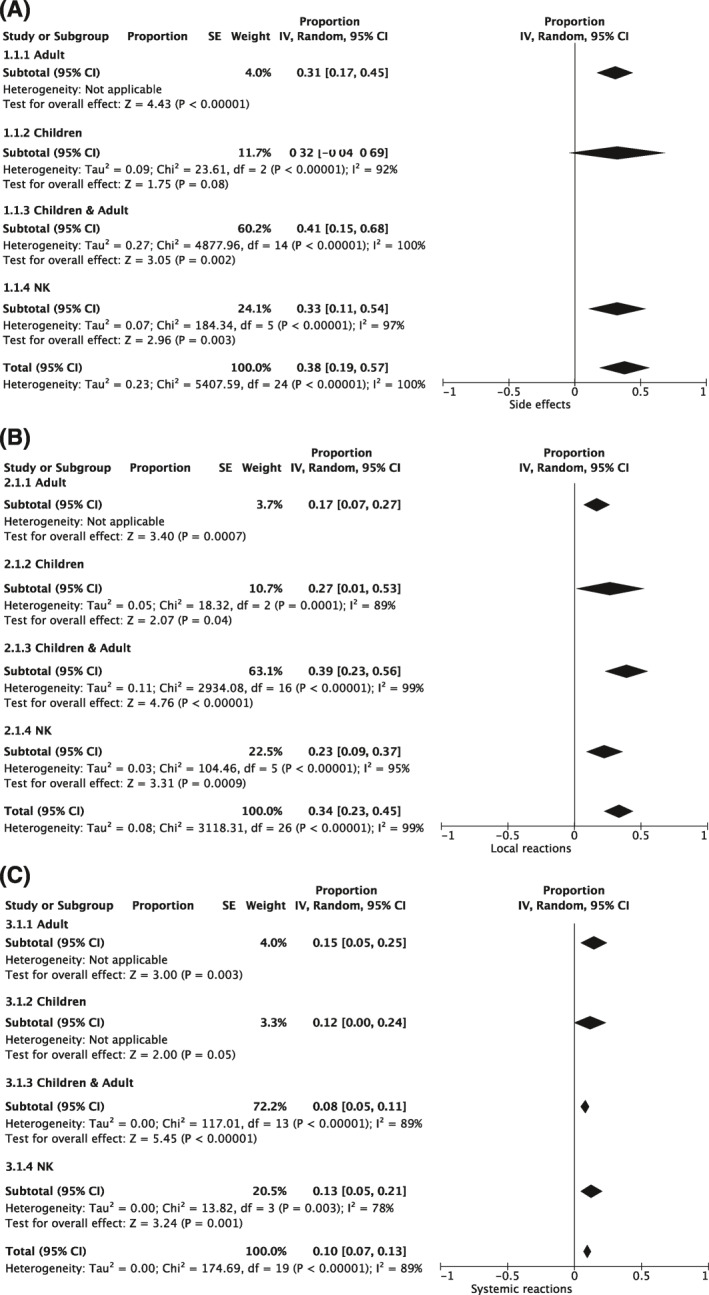
Subgroup analysis with regards to age: Overview of the meta‐analysis regarding (A) overall side effects (adult: *N* = 1 study, children: *N* = 3 studies, children and adult: *N* = 15 studies, NK: *N* = 6 studies), (B) local (adult: *N* = 1 study, children: *N* = 3 studies, children and adult: *N* = 17 studies, NK: *N* = 6 studies), and (C) systemic reactions (adult: *N* = 1 study, children: *N* = 1 study children and adult: *N* = 14 studies, NK: *N* = 4 studies). The random effects model was applied with inverse variance (IV) for study weight. Results are displayed as proportion of patients with side effects or local/systemic reactions, 95% CI (confidence interval) as well as analysis of heterogeneity. Further details are displayed in Additional file 6

Three studies were evaluable with regard to the safety of MATA treatment in children and adolescents.^33^, ^55^, ^57^ One study included only adults,^37^ 17 studies included both, adults and children/adolescents^15^, ^17^, ^25^, ^26^, ^28^, ^29^, ^30^, ^35^, ^40^, ^42^, ^44^, ^45^, ^46^, ^49^, ^51^, ^56^, ^60^ and in 6 studies^31^, ^36^, ^38^, ^54^, ^58^, ^59^ the age group is not known (NK, Table 3A). Analysis of any side effects (local and systemic) showed that nearly 32% of the children/adolescents experienced side effects. In comparison, in one study that included only adults, 31% of the patients experienced side effects. Interestingly, in the studies including children/adolescents and adults, side effects occurred in 41% of the patients. In the studies where the age‐group is not known, 33% of the patients experienced side effects (Figure 6A, Additional file 7A).

**TABLE 3:** Overview of the total number of patients treated with MATA in the subgroups with regards to (A) age and (B) asthma and the number of patients who experienced side effects or local/systemic reactions

*N* patients	Total/with side effects	Total/with local reactions	Total/with systemic reactions
(A) Subgroup with regards to age			
Only adults	48/15	48/8	48/7
Only children/adolescents	51/20	51/18	51/3
Adults and children/adolescents	2739/451	2865/488	932/89
Age group unknown	470/160	470/110	470/48
(B) Subgroup with regards to asthma			
Only asthmatics	171/38	171/31	171/7
Only non‐asthmatics	407/117	407/96	214/28
Asthmatics and non‐asthmatics	2509/407	765/238	2493/107
Asthma status unknown	221/84	221/106	158/28

When breaking this down to local and systemic reactions, 27% of the children and adolescents experienced local reactions and 12% systemic reactions. In comparison, 17% of the patients in the only‐adult study and 39% in the mixed‐age studies had local reactions (Figure 6B, Additional file 7B). Systemic reactions occurred in 15% of the patients in the study with adults and in 8% of the patients in the studies with adults and children/adolescents. In patients where the age group is not known, 23% experienced local reactions and 13% experienced systemic reactions (Figure 6C, Additional file 7C).

#### 3.8.2 Asthmatic patients

**FIGURE 7:**
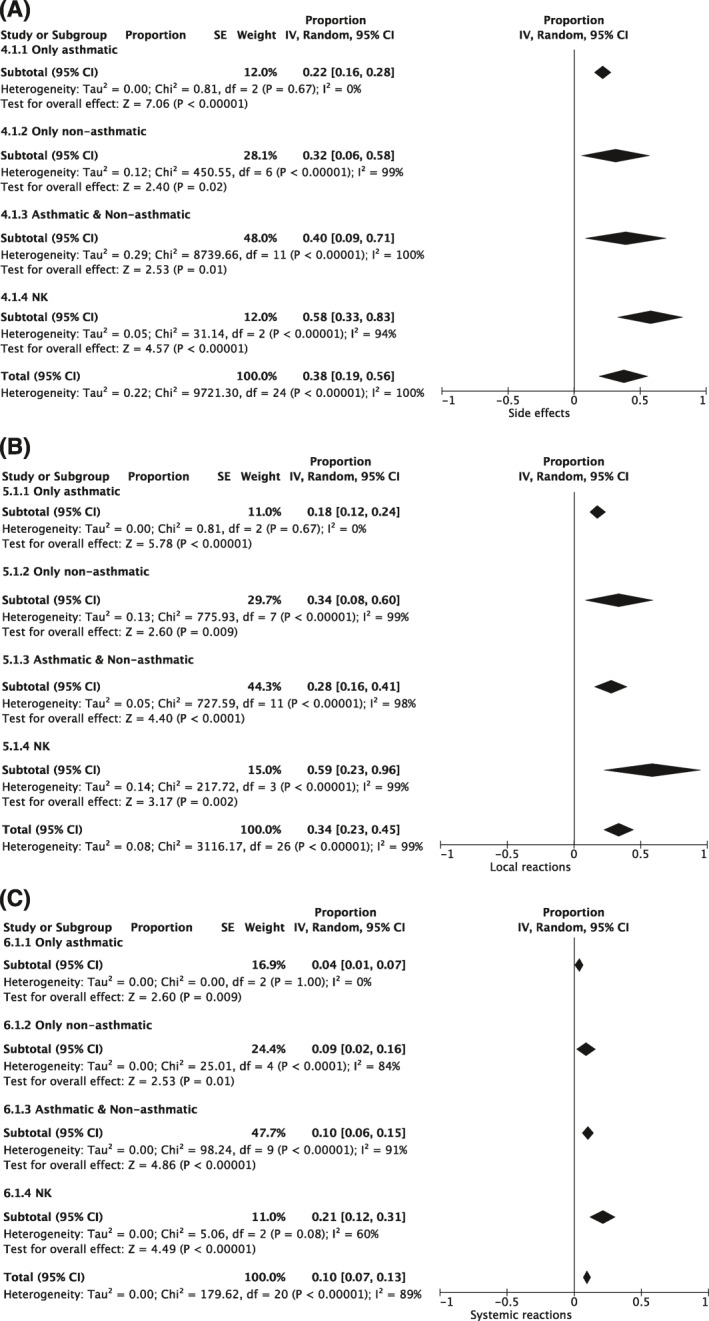
Subgroup analysis with regard to asthma status. Overview of the meta‐analysis regarding (A) overall side effects (only asthmatic: *N* = 3 studies, only non‐asthmatic: *N* = 7 studies, asthmatic & non‐asthmatic: *N* = 12 studies, NK: *N* = 3 studies), (B) local (only asthmatic: *N* = 3 studies, only non‐asthmatic: *N* = 8 studies, asthmatic & non‐asthmatic: *N* = 12 studies, NK: *N* = 4 studies) and (C) and systemic reactions (only asthmatic: *N* = 3 studies, only non‐asthmatic: *N* = 5 studies, asthmatic & non‐asthmatic: *N* = 10 studies, NK: *N* = 3 studies). The random effects model was applied with inverse variance (IV) for study weight. Results are displayed as proportion of patients with side effects or local/systemic reactions, 95% CI (confidence interval) as well as analysis of heterogeneity. Further details are displayed in Additional file 7

Three studies^26^, ^40^, ^45^ included only asthmatic and eight studies^15^, ^33^, ^35^, ^38^, ^44^, ^51^, ^54^, ^59^ only non‐asthmatic patients. Twelve studies^17^, ^28^, ^29^, ^31^, ^36^, ^42^, ^46^, ^49^, ^55^, ^56^, ^57^, ^60^ included both, asthmatic and non‐asthmatic patients and for four studies,^25^, ^30^, ^37^, ^58^ the asthma status is not known (NK, Table 3B). Any side effects (local and systemic) occurred in 22% of the asthmatic patients. In comparison, 32% of non‐asthmatics and 40% of patients included in the asthmatics/non‐asthmatics studies experienced side effects. Of the patients for whom the asthma status was not known, 58% experienced side effects (Figure 7A, Additional File 8A). More detailed, 18% of asthmatic patients experienced local reactions and 4% experienced systemic reactions. In the studies with only non‐asthmatics and both (asthmatics as well as non‐asthmatics), 34% and 28% of the patients, respectively, experienced local reactions. Systemic reactions occurred in 9% of the non‐asthmatic and in 10% of the mixed population (asthmatic/non‐asthmatic patients). Of the patients with unknown asthma status, 59% experienced local reactions and 21% experienced systemic reactions (Figure 7B, C, Additional file 8B, C).

## 4 DISCUSSION

This meta‐analysis demonstrates that AIT with MATA significantly improves allergic symptoms and reduces the use of allergic medication, resulting in a clinically meaningful treatment effect in patients with allergic rhinoconjunctivitis. Furthermore, subgroup analyses revealed that the treatment is also safe in vulnerable patient groups like children/adolescents and asthmatics. These results support the conclusions drawn from earlier DBPCTs. They are not only in line with, but even better than the results found in the largest meta‐analysis for AIT to date, which included studies in which subcutaneous and sublingual routes of application were assessed.^2^ Regarding the clinical efficacy of MATAs, represented by the CSMS, in comparison to this largest meta‐analysis, the SMD in the study presented here was −0.80, being 1.6‐fold better than in the study by Dhami et al., where the SMD was −0.49. While the SMD of the TMS was −0.38 in the large meta‐analysis,^2^ the analysis of MATA DBPCTs revealed an SMD of −2.20, being more than six times better. For the TSS, the SMD after MATA treatment was −1.20, whereas in the large meta‐analysis,^2^ the SMD for the TSS was −0.53, showing that MATA was 2.3‐fold better. However, it must be noted that these 160 studies included in the meta‐analysis by Dhami et al. investigated all kinds of AIT, like native and modified allergens, different allergen sources (pollen, mites, animal dander, etc.) and mode of application (e.g., subcutaneous, sublingual, intralymphatic immunotherapy). Due to this heterogeneity of this particular analysis only general statements could be drawn, in such that AIT is effective in improving allergic symptoms and reduces symptomatic medication intake.

In contrast, another publication restricted the meta‐analysis to grass pollen products for subcutaneous and sublingual AIT^62^ to minimize heterogeneity. The SMD of the CSMS following subcutaneous AIT in this study was −0.32, being not as good as in the meta‐analysis by Dhami et al. (SMD −0.49), and being 60% lower than the SMD in our study. Similar differences were also detected for the total medication score with −0.20 in contrast to an SMD of −2.20 determined in our study. The SMD of the symptom score was also four‐times higher in the MATA studies (−1.20) in comparison to the SMD evaluated in the grass‐pollen‐specific meta‐analysis (−0.27).^62^ However, in the meta‐analysis by Nelson et al., no information is given on the evaluated products. To our knowledge, there is currently only one published product‐line specific meta‐analysis, evaluating efficacy and safety of depigmented‐polymerized allergen extracts (DPAEs),^63^ In this study, the primary endpoint and safety endpoints were similar to the endpoints in our study. The SMD of the cSMS was in favor of DPAEs as was the SMD of the total symptom score. Data regarding medication scores of DPAEs in comparison to placebo were not given in the meta‐analysis by Mösges et al.

Comparison of the SMDs of the 4 studies (−0.49^2^, −0.32^62^, 1.9^63^, and −0.80 in our study) shows that product(‐line) specific meta‐analyses give a much more differentiated view of AIT products, whereas large meta‐analyses give a good overview about AIT in general but lack specificity.

Since the studies analyzed here are from the 1970s to 1990s, the CSMS used in these DBPCTs cannot be identical as the CSMS published in the EAACI position paper from 2014.^64^ However, we merged data in analogy to the CSMS of the position paper. As we were able to harmonize the symptom and medication scores of the studies analyzed here with a CSMS published 30–40 years later shows that the included MATA studies were state of the art studies, when conducted. These studies were prospective randomized and controlled studies, but the endpoints differed from the endpoints defined nowadays. However, by translation of the original medication and symptom scores into current CSMS standards we unraveled convincing state‐of‐the‐art evidence for the efficacy of MATA by this meta‐analysis.

Evaluation of the quality of the DBPC studies using the Jadad score—that was published in 1996—revealed that only two of the eight studies lacked sufficient quality.^16^, ^17^ The six remaining studies scored three points or more.^13^, ^14^, ^15^, ^18^, ^19^, ^20^ Three of the analyzed studies^14^, ^15^, ^18^ got the full score (five points), showing that studies from the mid‐1970s were already conducted according to current standards by applying a randomized, double‐blind, placebo‐controlled study design, resulting in a high quality of study according to a rating system that was developed 20 years later.

The rather low number of appropriate DBPCTs is explained by different historic endpoints. For instance, some studies focused on symptom or medication scores only, whereas others only evaluated changes in allergen specific IgG. With exception of sIgG we did not focus on biomarker analyses in this meta‐analysis. However, in some studies,^13^, ^14^, ^15^, ^16^ allergen specific IgE was also evaluated, but the data presented in these publications were not suitable for meta‐analysis (e.g., because of missing SD or SEM, no data on the outcome in the placebo group, sIgE only determined at screening visit). At the time when the included studies were carried out (from 1974 to 1995), some biomarkers—like IgG4 or the facilitated allergen binding assay (FAB) —were still unknown or their influence on the immune response following AIT had not yet been (fully) investigated or developed. Due to these missing parameters, Gelhar et al.^65^ published an approach to monitor AIT treatment by analyzing allergen‐specific IgG4/IgG1 ratios. This study was published in 1999, 4 years after the most recent study included here was published.^20^ The use of the FAB assay as a method to detect inhibitory antibody responses, was published in 2006 by Shamji et al.^66^—more than 10 years after the study of Rozniecka et al.^20^ In addition to the lack of biomarkers in the time frame of the studies presented here, other meta‐analyses, like the large meta‐analysis by Dhami et al.,^2^ Mösges et al.^63^, and Nelson et al.^63^ focused on patient related outcomes with a CSMS as primary endpoint. Biomarkers are important, but in our opinion, patient related outcomes, like the CSMS, are even more important, because above all, the patient must experience a symptom relief (in line with reduced symptomatic medication use).

While 8 studies could be included in the efficacy analysis, 43 studies could be evaluated with regards to overall safety, as well as local and systemic reactions. In comparison to the large meta‐analysis from 2017,^2^ where studies needed to be at least double‐blind to be included for safety analysis, the rate ratio was 2.37 for patients experiencing a systemic AE and 1.72 for patients experiencing a local reaction. In the study presented here, the proportion for the occurrence of local reactions was 34% and 10% for systemic reactions in the safety studies. However, for the study presented here, the proportion was calculated, whereas in the large meta‐analysis by Dhami et al., rate ratios were calculated. Despite the high possibility of developing immediate local reactions following MATA treatment in comparison to the large meta‐analysis, the risk of developing systemic reactions was similar in both meta‐analyses. When focusing on the DBPC studies, the risk of developing local reactions after MATA treatment is considerably higher (RR for immediate local reactions: 11.61 [CI: 2.09, 64.59]) than in comparison to the large meta‐analysis.^2^ With regard to systemic reactions, the RR was also higher (6.43 [CI: 1.14, 36.30]). However, only two or rather three studies were analyzable with regard to local and systemic reactions, following MATA treatment. Whereas in the large meta‐analysis by Dhami et al., 9 studies were analyzed with regard to local and 15 studies with regard to systemic reactions, with a heterogeneity index I^2^ of 64% and 83% (for systemic reactions).

In addition to the overall safety analysis, special emphasis was laid on asthmatics and children/adolescents in our meta‐analysis. Asthmatic patients are considered to be prone to side effects^67^, ^68^ and we therefore analyzed this subgroup with regards to side effects (local and systemic reactions). The analysis showed that there were no differences in the safety profile in asthmatic in comparison to non‐asthmatic patients and the overall patient collective. Interestingly, asthmatic patients experienced considerably fewer side effects and local/systemic reactions in comparison to non‐asthmatics and studies with a mixed patient population (asthmatics and non‐asthmatics). Before the turn of the millennium, asthmatic patients were usually treated with oral glucocorticosteroids, which can contribute to avoiding/reducing side effects and local/systemic reactions. In a previous product‐specific analysis by Mösges et al. with polymerized allergen extracts, it was also shown that asthma is not a risk factor when treating patients with AIT.^63^

Since MATAs are licensed for patients aged 5 years and older, some studies also included children and adolescents. This is a vulnerable patient population and therefore constituted an additional focus in our meta‐analysis. Here, no differences with regard to side effects, local or systemic reactions were detected when comparing the studies including children and adolescents with the only‐adult study or the studies including patients of all ages who were treated with MATA. These results are particularly interesting for pediatricians, as they show that children can be treated safely with MATAs. With this early treatment option, the progression to allergic asthma can be considerably inhibited, if not prevented entirely, as was shown by Möller at al.^69^ In this study, asthma symptoms of children who were treated with immunotherapy improved significantly in comparison to the placebo group, highlighting the importance of AIT already in childhood.

The analysis presented here is based on the first year in case of 2‐years studies. This is due to the fact that in many studies included in the meta‐analysis, the treatment and endpoints were changed after the first year.

It may be considered as a limitation, that this product‐line specific meta‐analysis was not split with regard to the single allergens or allergen‐mixtures.^3^ Although this is recommended in the WAO statement, we believe it is sufficient to test the product line with the different allergens and allergen‐mixtures, as we mainly aimed to evaluate the efficacy and safety of MATAs, independent of the allergen. Furthermore, the allergens evaluated in this meta‐analysis are solely derived from pollen (trees, grasses, weed), which provides a certain homogeneity. Therefore, despite the use of different allergens in the analyzed studies, we did not perform allergen‐specific subgroup analyses.

A similar approach (product‐line specific meta‐analyses without allergen‐specific subgroup analyses) was already done by Mösges et al.^63^ In this study, the authors showed that the heterogeneity of the results was due to the underlying severity of disease, rather the different allergens, demonstrating the comparability of the different allergens within the product.

## 5 CONCLUSION

The meta‐analysis presented here demonstrates that a large body of evidence exists for AIT with glutaraldehyde‐modified and MCT^®^‐adsorbed allergen extracts. MATA treatment significantly improved allergic symptoms and reduced the use of anti‐allergic medication in allergic patients in comparison to placebo, going in line with a good safety profile.

Looking especially at children and asthmatic patients, the use of MATA can be considered safe, as their safety profiles did not differ from the overall patient population.

## CONFLICT OF INTEREST

SB reports personal fees from Allergopharma, ALK, HAL Allergy, Thermo Fischer Scientific, Sanofi Genzyme, Novartis, Bristol Meyers Squibb, MSD, Mylan, grants and personal fees from Bencard, Storz GmbH, BRAIN AG outside the submitted work. PZ reports grants and personal fees from Alk Abello, grants and personal fees from Allergopharma, grants and personal fees from Allergy Therapeutics, personal fees from Bencard, grants from Biomay, grants from Calistoga, grants from GSK, grants and personal fees from HAL, grants and personal fees from Marinomed, personal fees from Leti, personal fees from Meda, personal fees from Merck, grants from MSD, personal fees from Novartis, grants from Ono, grants from Oxagen, grants from RespiVert, personal fees from Sigmapharm, grants and personal fees from Stallergenes, personal fees from Thermo Fisher Scientific, grants from VentirX, all outside the submitted work. GC has nothing to disclose. FF received personal fees from Allergy Therapeutics Italia. PY has nothing to disclose. EHSK reports personal grant from Bencard, travel support from ALK, personal grant from Vifor. CA has nothing to disclose. ER reports personal fees from Bayer and personal fees from Gesellschaft für Phytotherapie outside the submitted work. SA reports grants and personal fees from Lofarma, personal fees from Servier, personal fees from Hexal, personal fees from Friulchem, outside the submitted work. RM reports personal fees from ALK, grants from ASIT biotech, personal fees from allergopharma, personal fees from Allergy Therapeutics, grants and personal fees from Bencard, grants from Leti, grants, personal fees and non‐financial support from Lofarma, non‐financial support from Roxall, grants and personal fees from Stallergenes, grants from Optima, personal fees from Friulchem, personal fees from Hexal, personal fees from Servier, personal fees from Klosterfrau, non‐financial support from Atmos, personal fees from Bayer, non‐financial support from Bionorica, personal fees from FAES, personal fees from GSK, personal fees from MSD, personal fees from Johnson&Johnson, personal fees from Meda, personal fees and non‐financial support from Novartis, non‐financial support from Otonomy, personal fees from Pohl‐Boscamp, personal fees from Stada, personal fees from Hikma, personal fees from UCB, non‐financial support from Ferrero, grants from BitopAG, grants from Hulka, personal fees from Nuvo, grants from Ursapharm, outside the submitted work.

## AUTHOR CONTRIBUTIONS

Sven Becker: Funding Acquisition (lead), Conceptualization (supporting), Methodology (equal), Supervision (supporting), Validation (lead), Writing – review and editing (supporting). Petra Zieglmayer: Writing – review and editing (supporting). Gabriela Canto: Writing – review and editing (supporting). Filippo Fassio: Writing – review and editing (supporting). Patrick Yong: Writing – review and editing (supporting). Cengizhan Acikel: Formal Analysis (lead), Visualization (equal). Esther Raskopf: Data Curation (supporting) Formal Analysis (supporting), Visualization (equal), Writing ‐ original draft preparation (lead), Writing – review and editing (lead). Esther Helen Steveling‐Klein: Writing – review and editing (supporting). Silke Allekotte: Conceptualization (equal), Methodology (equal), Data Curation (lead), Visualization (supporting), Writing – original draft preparation (supporting), Writing – review and editing (supporting). Ralph Mösges: Conceptualization (equal), Methodology (equal), Supervision (lead), Writing – review and editing (supporting).

## Supporting information

Supplementary Material

Supplementary Material

Supplementary Material

Supplementary Material

Supplementary Material

Supplementary Material

Supplementary Material

Supplementary Material
