# Supplementary material for: A meta‐analysis on allergen‐specific immunotherapy using MCT® (MicroCrystalline Tyrosine)‐adsorbed allergoids in pollen allergic patients suffering from allergic rhinoconjunctivitis
Source: Clin Transl Allergy. 2021 Jun 3;11(4):e12037. doi: 10.1002/clt2.12037 (PMC8174800; doi:10.1002/clt2.12037)
Supplement: Supplementary file 1 — Supplementary Material [file CLT2-11-e12037-s005.docx]

**Additional File 1: Literature search**

**Search syntax in Medline database:**

| **Advanced search** |
| --- |
| ((((“pollen” [MeSH Terms]) OR (“pollen” [All Fields])) AND (“tyrosine” [MeSH Terms])) OR (“tyrosine” [All Fields])) AND (“clinical trial” [Publication Type]) |
| ((((“poaceae” [MeSH Terms]) OR (“poaceae” [All Fields])) OR (“grass” [All Fields])) AND (“pollen-tyrosine” [All Fields])) AND (“adsorbate” [All Fields]) |
| (((((“dust” [MeSH Terms]) OR (“dust” [All Fields])) OR (“house” [All Fields])) AND (“dust” [All Fields])) OR (“house dust” [All Fields])) AND (“adsorbate” [All Fields]) |
| ((((((((“Parietaria judaica pollen” [Supplementary Concept]) OR (“Parietaria judaica pollen” [All Fields])) OR (“pollinex” [All Fields])) AND (“rhinitis, allergic, seasonal” [MeSH Terms])) OR (“rhinitis” [All Fields])) AND (“allergic” [All Fields])) AND (“seasonal” [All Fields])) OR (“seasonal allergic rhinitis” [All Fields])) OR (“hayfever” [All Fields]) |
| (“desensitization, immunologic” [MeSH Terms]) AND (“tyrosine allergoid” [All Fields]) |
| **Basic search** |
| glutaraldehyde-modified pollen-tyrosine adsorbate |
| glutaraldehyde modified ragweed tyrosine adsorbate |
| glutaraldehyde-modified tyrosine-adsorbed immunotherapy |
| tyrosine adsorbed pollen extract |
| grass pollen-tyrosine adsorbate |
| tyrosine allergoids |

**Search syntax in Latin American and Caribbean Literature on Health Sciences (LILACS) database:**

| tw:(Pollinex)) AND (tw:(immunotherapy) |
| --- |

**Search syntax in in ZB MED Search Portal for Life Sciences (LIVIVO)**

| DT=(clinical trial) AND (specific immunotherapy) AND Pollinex |
| --- |
| tyrosine-absorbed AND immunotherapy |
| immunotherapy AND modified tyrosine adsorbate |
| immunotherapy AND tyrosine adsorbate NOT Quattro |
| immunotherapy AND glutaraldehyde-modified AND tyrosine adsorbed |

**Search syntax Web of Science**

| clinical trial [topic] AND specific immunotherapy [topic] AND Pollinex [topic] |
| --- |
| tyrosine-absorbed [topic] AND immunotherapy [topic] |
| immunotherapy [topic] AND modified tyrosine adsorbate [topic] |
| immunotherapy [topic] AND tyrosine adsorbate [topic] NOT Quattro [topic] |
| immunotherapy [topic] AND glutaraldehyde-modified [topic] AND tyrosine adsorbed [topic] |

**Search Syntax in Google and Google Scholar:**

| Depot-Pollenextrakt (L-Tyrosin-Adsorbat) |
| --- |
| TA Tyrosin-Allergoid |
