# Supplementary material for: A meta‐analysis on allergen‐specific immunotherapy using MCT® (MicroCrystalline Tyrosine)‐adsorbed allergoids in pollen allergic patients suffering from allergic rhinoconjunctivitis
Source: Clin Transl Allergy. 2021 Jun 3;11(4):e12037. doi: 10.1002/clt2.12037 (PMC8174800; doi:10.1002/clt2.12037)
Supplement: Supplementary file 2 — Supplementary Material [file CLT2-11-e12037-s003.docx]

**Additional File 2: Definition of responders and non-responders in the DBPC studies.**

| **Study** | **Evaluation by** | **Definition of responder** | **Definition of non-responder** |
| --- | --- | --- | --- |
| Miller ^18^ | patients | response to treatment good/very good | response to treatment not good |
| Adamek-Guzik ^17^ | unknown | improvement | no change, deterioration |
| Weisnagel ^16^ | parents of the patient | a bit better/better by 50% or more | no change, deterioration |
| Cockroft ^14^ | patients | good/very good | poor |
| Mischler ^15^  (both seasons) | physician | good (>50% improvement) | poor (<50% improvement) |
