# Supplementary material for: A meta‐analysis on allergen‐specific immunotherapy using MCT® (MicroCrystalline Tyrosine)‐adsorbed allergoids in pollen allergic patients suffering from allergic rhinoconjunctivitis
Source: Clin Transl Allergy. 2021 Jun 3;11(4):e12037. doi: 10.1002/clt2.12037 (PMC8174800; doi:10.1002/clt2.12037)
Supplement: Supplementary file 4 — Supplementary Material [file CLT2-11-e12037-s002.docx]

**Additional File 4: single scores – Forest and Funnel plots**

Meta-analysis and Funnel plots of the DBPCTs with regard to the single scores A) sneezing, B) nasal obstruction, C) eye symptoms and D) coughing in patients treated with MATA and placebo treated patients at the time of the primary analysis in the respective Studies. The random effects model was applied with inverse variance (IV) for study weight. Results are displayed as standardized mean difference with 95% CI (confidence interval) as well as analysis of heterogeneity. The studies are presented with N of patients, mean and SD (standard deviation). Publication bias is displayed using Funnel plots.

A) Sneezing

B) Nasal obstruction

C) Eye symptoms

D) Coughing
