# Supplementary material for: A meta‐analysis on allergen‐specific immunotherapy using MCT® (MicroCrystalline Tyrosine)‐adsorbed allergoids in pollen allergic patients suffering from allergic rhinoconjunctivitis
Source: Clin Transl Allergy. 2021 Jun 3;11(4):e12037. doi: 10.1002/clt2.12037 (PMC8174800; doi:10.1002/clt2.12037)
Supplement: Supplementary file 5 — Supplementary Material [file CLT2-11-e12037-s001.docx]

**Additional File 5: Safety analyzed in the DBPC studies**

Meta-analysis and Funnel plots of the DBPCTs comparing A) immediate and B) late local reactions as well as C) systemic reactions per injection in patients treated with MATA and placebo treated patients at the time of the primary analysis in the respective Studies. The random effects model was applied with inverse variance (IV) for study weight. Results are displayed as risk ratio (RR) with 95% CI (confidence interval) as well as analysis of heterogeneity. The studies are presented with N of patients, and N of events (= patients who improved after treatment with MATA). Publication bias is displayed using Funnel plots.

A) Immediate local reactions per injection

B) Late local reactions per injection

C) Systemic reactions per injection
