# Supplementary material for: A meta‐analysis on allergen‐specific immunotherapy using MCT® (MicroCrystalline Tyrosine)‐adsorbed allergoids in pollen allergic patients suffering from allergic rhinoconjunctivitis
Source: Clin Transl Allergy. 2021 Jun 3;11(4):e12037. doi: 10.1002/clt2.12037 (PMC8174800; doi:10.1002/clt2.12037)
Supplement: Supplementary file 6 — Supplementary Material [file CLT2-11-e12037-s006.docx]

**Additional File 6: Funnel plots of CSMS, TSS, TMS, sIgG and improvement of allergic condition**

Analysis of publication bias of the primary efficacy parameters CSMS (A), TSS (B), TMS (C), sIgG (D) and improvement of allergic condition (E).

A) CSMS

B) TSS

C) TMS

D) sIgG

E) Improvement
