# Supplementary material for: A meta‐analysis on allergen‐specific immunotherapy using MCT® (MicroCrystalline Tyrosine)‐adsorbed allergoids in pollen allergic patients suffering from allergic rhinoconjunctivitis
Source: Clin Transl Allergy. 2021 Jun 3;11(4):e12037. doi: 10.1002/clt2.12037 (PMC8174800; doi:10.1002/clt2.12037)
Supplement: Supplementary file 8 — Supplementary Material [file CLT2-11-e12037-s007.docx]

**Additional file 8: Safety with regard to asthma**

Subgroup meta-analysis concerning asthma status of the patients: Detailed overview of the meta-analysis and Funnel plots regarding A) overall side effects, B) local and C) and systemic reactions. The random effects model was applied with inverse variance (IV) for study weight. Results are displayed as proportion of patients with side effects or local/systemic reactions, 95% CI (confidence interval) as well as analysis of heterogeneity. Publication bias is displayed using Funnel plots.

A) Proportion of side effects by asthma status

B) Proportion of local reactions by asthma status

C) Proportion of systemic reactions by asthma status
